# Effects of *Heyndrickxia coagulans* on Waterborne Copper Toxicity

**DOI:** 10.3390/life15020300

**Published:** 2025-02-14

**Authors:** Chung-Tsui Huang, Chao-Yi Chen, Yao-Jen Liang

**Affiliations:** 1 Department of Internal Medicine, Division of Gastroenterology and Hepatology, Far Eastern Memorial Hospital, New Taipei City 220, Taiwan; 0989206131ng@gmail.com; 2 Graduate Institute of Applied Science and Engineering, Fu-Jen Catholic University, New Taipei City 242, Taiwan; g730325@hotmail.com; 3 Department and Institute of Life Science, Fu-Jen Catholic University, New Taipei City 242, Taiwan

**Keywords:** ocean pollution, waterborne copper toxicity, *Heyndrickxia coagulans*, biosorption, heavy metal

## Abstract

Copper contamination in coastal water environments poses a significant health risk, and traditional treatments for copper intoxication include gastric lavage, chelation, and hemodialysis. Recent research suggests that probiotics may help mitigate heavy metal toxicity by promoting biosorption in the intestinal tract. To explore this potential, we investigated the protective effects of *Heyndrickxia coagulans* (*H. coagulans*) against copper-induced toxicity in rats. After eight weeks of exposure, rats receiving both copper and *H. coagulans* exhibited significant improvements in renal function, lipid profiles, antioxidant enzyme activity, and histological markers compared to the copper-only group. However, liver function remained largely unchanged, suggesting a more pronounced protective effect on renal health. These findings highlight the potential of *H. coagulans* as a supportive intervention for mitigating the adverse effects of copper intoxication.

## 1. Introduction

Waterborne heavy metals are common toxic agents in modern life. These heavy metals can enter the human body through the oral route when individuals consume copper-contaminated water or fish [1,2]. Copper is a common agent of heavy metal toxicity and has the potential to cause organ injury. Exposure to excessive copper can lead to hepatic dysfunction with manifestations similar to Wilson’s disease, an inherited disorder caused by impaired copper excretion from hepatocytes due to mutations in the ATP7B gene, which encodes a hepatic enzyme responsible for the transmembrane transport of copper [3,4,5].

The severe symptoms or signs of copper poisoning primarily include hepatitis, with clinical presentations such as nausea, anorexia, jaundice, and the gradual deposition of copper in the cornea, forming a characteristic sign called the Kayser–Fleischer ring. The treatment of copper toxicity consists of three primary methods: gastric lavage, chelation therapy, and hemodialysis. The choice of therapeutic method depends on whether the poisoning is acute or chronic, as well as on laboratory evaluations of severity. However, there is a lack of therapeutic approaches targeting the intestinal lumen to reduce copper absorption into the bloodstream.

*Heyndrickxia coagulans* (Hammer 1915) Narsing Rao et al., 2023 is a Gram-positive, facultatively anaerobic, and neutral bacterium classified as a lactic acid-forming species within the Bacillus genus [6]. It exhibits a cylindrical cell shape with blunt ends, appearing singly, in pairs, or occasionally in short chains. The bacteria produce spores at the tips and lack flagella. Optimal growth conditions range from 30 to 50 °C with a pH of 5.5–6.5 [7]. *H. coagulans* ferments various substrates, including maltodextrin, mannitol, raffinose, sucrose, and trehalose, producing acid without gas. A distinguishing characteristic is its oval endospores, located at the bacterial pole, in contrast to the central or subterminal positioning seen in other Bacillus species [8]. The median genome size of *H. coagulans* is 3.42411 Mb, with an average of 3190 proteins and a GC content of 46.5%. It is further characterized by the absence of cytochrome c oxidase and its inability to reduce nitrate to nitrite. Different strains of *H. coagulans* have varied growth factor requirements, often necessitating biotin and thiocyanin, which they cannot synthesize independently [8]. Its spores exhibit strong resistance to gastric acid, enabling them to germinate in the small intestine within 4–6 h after ingestion. However, due to weak adhesion to intestinal epithelial cells, these bacteria typically remain transient in the gut, being eliminated through defecation within 4–7 days [9]. To maximize its probiotic benefits, *H. coagulans* must be consumed regularly. Its spores demonstrate high-stress resistance, withstanding extreme temperatures, acidity, and bile salts, which allows it to survive where other probiotics fail and germinate at the optimal time [10]. This advantage mitigates the limitations of conventional probiotics. *H. coagulans* is widely applied in aquaculture as an antibiotic alternative, in medical fields for probiotic tablet production, and in the food and healthcare industries. Its beneficial effects stem from its ability to regulate physiological and chemical properties, promoting beneficial bacteria while inhibiting harmful ones, thereby improving immune function [11].

*H. coagulans* exhibits weak adhesion to intestinal epithelial cells, preventing long-term colonization. As a result, the bacteria are typically cleared from the gut within 4–7 days through defecation [12]. This transient nature necessitates continuous supplementation, which may affect patient compliance. The growth of *H. coagulans* is dependent on specific temperature and humidity conditions, as well as essential growth factors like biotin and thiamine, which it cannot synthesize on its own. This dependency can limit its viability and effectiveness in certain applications. The bacterium lacks key enzymes such as cytochrome C oxidase, restricting its functionality in some biological processes [13]. Although *H. coagulans* spores are highly resistant to high temperatures, acidity, and bile salts, they still require specific conditions to germinate and become active. This may reduce their effectiveness in mild environments or in individuals with different gut conditions.

For heavy metal toxicity, *H. coagulans* and *Lactiplantibacillus plantarum* have been shown to have protective effects on hepatic and renal functions in rats with mercury intoxication or acute cadmium toxicity [14]. Additionally, *Lactiplantibacillus plantarum* (CCFM8661) has been studied for its ability to alleviate lead toxicity in a mouse model [15].

Theoretically, probiotics can prevent copper from being absorbed into the bloodstream by competing with enterocytes through the biosorption mechanism [16,17]. Probiotics, such as *H. coagulans*, have been shown to be beneficial for mitigating certain heavy metal toxicities, such as those caused by lead and cadmium. However, there is a lack of animal studies investigating the potential of *H. coagulans* in ameliorating the toxic effects of copper, although recent studies have begun to address this gap [18,19,20]. Therefore, this study aimed to evaluate the therapeutic effects of *H. coagulans* on copper intoxication using a rat model.

## 2. Materials and Methods

### 2.1. Animal

Male adult Wistar rats were obtained from LASCO (BioLASCO Taiwan Co., Ltd., Yilan, Taiwan). Rats weighing approximately 250 g were used and housed in the Fu Jen Laboratory Animal Center. The environmental conditions were maintained as follows: temperature (21 ± 2 °C), humidity (55% ± 10%), a 12 h light/dark cycle, air exchange (10–15 times per hour), and noise levels (<65 dB). After a 1-week acclimatization period, the animals were randomly assigned to three groups.

The control group (group 1) was administered water daily via oral gavage. Group 2 was administered 10 mg/kg body weight (bw)/day copper dichloride (CuCl_2_; Sigma-Aldrich, St. Louis, MO, USA). Group 3 was administered 10 mg/kg bw/day copper dichloride along with 10.42 mg/kg bw/day *Heyndrickxia coagulans* TCI711 (*H. coagulans* TCI711). CuCl_2_ and *H. coagulans* were administered once daily. Both CuCl_2_ and *H. coagulans* were administered concomitantly. The treatment period lasted eight weeks, after which blood and tissue samples were collected for analysis. Each group consisted of six replicates (*n* = 6). *H. coagulans* TCI711 was provided as an experimental powder by TCI Co., Ltd. (Taipei, Taiwan). Rats in all groups were treated for 2 months and weighed individually twice a week.

All procedures involving animals in this study were conducted in accordance with the recommendations of the Institutional Animal Care and Use Committee (IACUC) of Fu Jen Laboratory Animal Center (FJU A10801).

### 2.2. Serum Biochemical Analysis

After centrifugation at 1500 g for 10 min, the serum was aliquoted into separator tubes. Biochemical parameters, including AST (aspartate transaminase), ALT (alanine transaminase), BUN (blood urea nitrogen), CREA (creatinine), TRIG (triglycerides), and CHOL (cholesterol), were measured using an IDEXX VetTest Chemistry Analyzer (IDEXX Laboratories, Westbrook, ME, USA).

### 2.3. The GSH and SOD Assays

Liver tissues were homogenized in cell lysis buffer (PRO-PREP™; iNtRON Biotechnology, Seongnam-si, Gyeonggi-do, Korea) according to the manufacturer’s instructions. GSH levels were measured using a Glutathione Assay Kit (Sigma-Aldrich, St. Louis, MO, USA), and SOD activity was measured using a Superoxide Dismutase Assay Kit (Sigma-Aldrich, St. Louis, MO, USA). Quantification relative to total protein was performed using a BCA Protein Assay Kit (Thermo Fisher Scientific, Waltham, MA, USA).

### 2.4. Liver Tissue Histopathology

Liver tissues were formalin-fixed and paraffin-embedded, and 4 µm thick paraffin sections were stained with hematoxylin and eosin (H&E) for copper quantification. Hepatitis and fibrosis scores were determined by a board-certified veterinary pathologist (TvdI) according to the WSAVA classification [21]. Copper accumulation (rubeanic acid; RA) was evaluated semi-quantitatively using a scale from 0 to 5, as previously described [21], and its localization within the liver lobule was assessed. 

### 2.5. Statistical Analysis

Statistical analysis to determine significant differences between group means was performed using two-way analysis of variance (ANOVA), followed by Duncan’s post hoc test with SPSS software (version 16.0). A *p*-value of <0.05 was considered statistically significant.

## 3. Results

### 3.1. Heyndrickxia coagulans (H. coagulans) Improves Renal Function and Lipid Metabolism in Copper Toxicity Models

**Figure 1:**
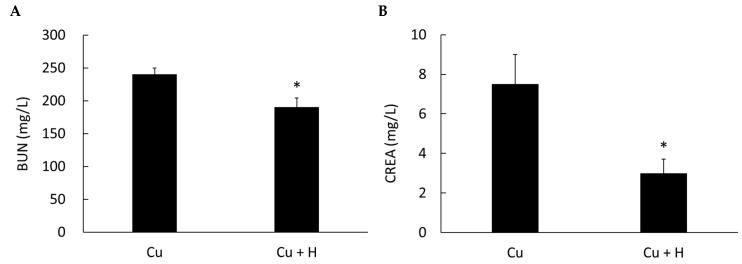
Comparison of blood urea nitrogen (BUN) and creatinine (CREA) levels between copper-poisoned rats fed with *H. coagulans* (Cu + H) and those without *H. coagulans* (Cu). A lower level of BUN and creatinine indicates better renal function. (**A**) BUN and (**B**) creatinine levels were significantly lower in the group fed *H. coagulans*. * *p* < 0.05 compared to the Cu group. (*n* = 6).

**Figure 2:**
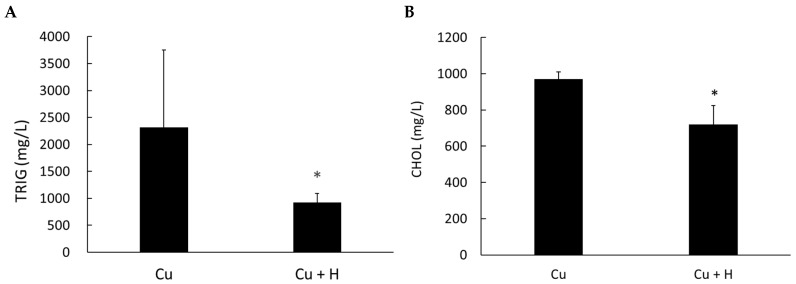
Comparison of triglyceride (TRIG) and total cholesterol (CHOL) levels between copper-poisoned rats fed with *H. coagulans* (Cu + H) and those without *H. coagulans* (Cu). (**A**) Triglyceride and (**B**) cholesterol levels were significantly lower in the group fed with *H. coagulans*. * *p* < 0.05 compared to the Cu group (*n* = 6).

In the group fed *H. coagulans*, AST and ALT levels were higher, but the difference was not statistically significant. However, renal function, as indicated by BUN and creatinine levels, was significantly improved, along with lipid factors such as total cholesterol and triglycerides (Figure 1 and Figure 2). These results suggest that *H. coagulans* is beneficial to kidney function in animals affected by copper toxicity and may be related to lipid absorption and metabolism in the intestine.

### 3.2. Protective Effects of H. coagulans on Liver and Kidney Histology in Copper Toxicity

**Figure 3:**
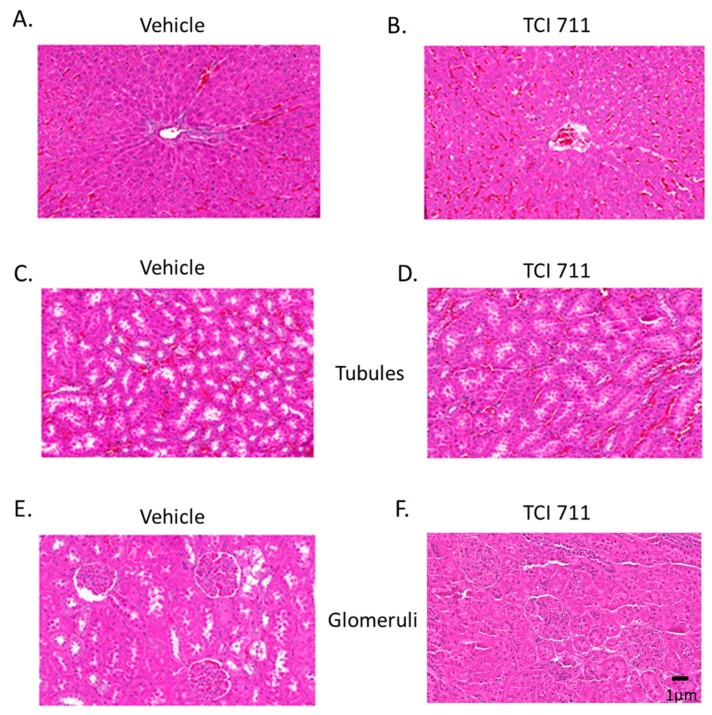
Comparison of liver and kidney histology between copper-poisoned rats fed with *H. coagulans* (TCI 117, right column) and those without *H. coagulans* (left column). Histological injuries were ameliorated in the group fed with *H. coagulans*.

To further understand the protective mechanism of *H. coagulans* against copper toxicity, we examined hepatic histology. Heavy metal exposure resulted in inflammatory cell infiltration, interstitial fibrosis, cytoplasmic vacuolization, and sinusoidal congestion. Renal histology showed proximal tubular changes and congestion. All these pathological changes were ameliorated in the *H. coagulans*-fed group (Figure 3, Table 1). These findings indicate that *H. coagulans* protects the liver and kidney from the cumulative toxicity of copper heavy metals and that the improvement in renal function is associated with structural protection of kidney tissue.

### 3.3. H. coagulans Enhances Liver Antioxidant Enzyme Activity

**Figure 4:**
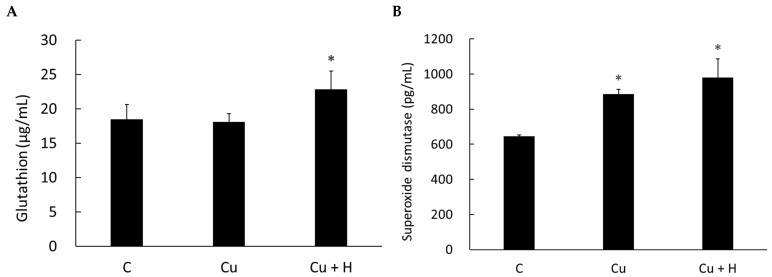
Comparison of glutathione and superoxide dismutase levels between group 1 (control, C), group 2 (copper, Cu), and group 3 (copper plus *H. coagulans*, Cu + H). (**A**) Glutathione and (**B**) superoxide dismutase levels were significantly higher in group 3. * *p* < 0.05 compared to the Cu group (*n* = 6).

An assessment of the glutathione (GSH) and superoxide dismutase (SOD) levels in liver tissue showed increased expression in the *H. coagulans*-fed group (Figure 4). These data demonstrate that *H. coagulans* effectively enhances the activity of liver antioxidant enzymes.

## 4. Discussion

Bacteria can remove heavy metals from the water environment through a process called biosorption [17,22]. Theoretically, biosorption is also feasible in the intestinal lumen of the digestive tract. If heavy metals are orally ingested and have not yet been absorbed by enterocytes, bacteria could compete with the enterocytes for these metals.

Copper poisoning in humans typically occurs through ingestion, with the gastrointestinal tract being the first organ system to show symptoms. In acute poisoning, patients present with nausea, diarrhea, and bleeding of the gastrointestinal mucosa. Once copper is absorbed into the bloodstream, dysfunction of internal organs, including the liver, kidneys, and bone marrow, can occur. Chronic poisoning develops more slowly, with common manifestations including neurological dysfunction, psychiatric issues, and endocrine disorders [23].

This study was designed to investigate whether *Heyndrickxia coagulans* (*H. coagulans*) could reduce waterborne copper toxicity when both are simultaneously present in the intestinal lumen of rats. It is reasonable to assume that a mixture of *H. coagulans* and copper in the intestinal luminal fluid would naturally facilitate biosorption.

Elevated AST and ALT levels are indicative of an acute inflammatory response in hepatocytes when damaged by heavy metals. Pathologically, AST and ALT levels do not increase proportionally, as only a small number of hepatocytes survive the external injury. In this study, the results showed higher, but non-significant, levels of AST and ALT in the group of rats fed *H. coagulans*. However, liver and kidney histology both demonstrated the protective effects of *H. coagulans* when copper toxicity was induced. This phenomenon can be explained by the fact that the number of surviving hepatocytes was higher in the *H. coagulans*-fed group, leading to an increase in AST and ALT levels. Furthermore, the quantitative assessment of two antioxidant enzymes, glutathione (GSH) and superoxide dismutase (SOD), was performed on liver tissue. The results showed that the *H. coagulans*-fed group exhibited increased levels of both enzymes. This phenomenon likely explains the reduced histological injuries in the liver of rats fed *H. coagulans*, due to the protective effects of antioxidants [24,25,26,27,28].

On the other hand, lipid factors in the blood also showed a positive response to *H. coagulans* feeding. It can be inferred that *H. coagulans* may interfere with the absorption of copper in the intestine, thereby reducing the accumulation of heavy metals in animals.

## 5. Conclusions

*Heyndrickxia coagulans* (*H. coagulans*) exhibits extensive potential applications in the biomedical field, particularly in alleviating kidney and liver injuries, regulating gut microbiota, and facilitating alcohol metabolism.

Firstly, *H. coagulans* may mitigate kidney and liver damage caused by waterborne copper poisoning through the gastrointestinal tract, demonstrating its potential for therapeutic applications. Additionally, this bacterial species may indirectly improve non-alcoholic fatty liver disease (NAFLD) by modulating gut microbiota. Studies have indicated that certain identified beneficial bacteria could be further developed into pharmaceuticals or health products, such as probiotics, postbiotics (metabolites of probiotics), and prebiotics (nutrients for probiotics) [29].

On the other hand, *H. coagulans* also shows promise in alcohol metabolism. A study revealed that endogenous alcohol dehydrogenase (ADH) and aldehyde dehydrogenase (ALDH) in this bacterial strain can facilitate alcohol metabolism while demonstrating resistance to gastric acid and bile salts. Further research confirmed that after a one-week intake, this bacterium enhances alcohol metabolism efficiency and accelerates alcohol clearance in the human body. For instance, experimental results showed that test subjects metabolized an amount of alcohol equivalent to 75 mL of whiskey within two hours. Consequently, *H. coagulans* holds potential for development into anti-alcoholic products [30].

In conclusion, *H. coagulans* demonstrates significant potential in treating kidney and liver injuries, regulating gut microbiota, and promoting alcohol metabolism. Future research should further explore its applications in biomedicine and functional foods to enhance human health.

## Figures and Tables

**Table 1:** Comparison of liver and kidney histology between group 1 (control), group 2 (copper), and group 3 (copper plus *H. coagulans*).

		Control	Cu	Cu + H
Liver	Inflammatory cell infiltration	None	Minimal	None
	Interstitial fibrosis	None	Minimal	None
	Sinusoidal congestion	None	Moderate	Mild
	Cytoplasmic vacuoles	None	Moderate (Peripheral)	Minimal (Peripheral)
	Necrosis	None	None	None
	Vessel damage	None	None	None
	Pigment deposition	None	None	None
	Fatty changes	Minimal (Microvesicular)	Minimal (Microvesicular)	Minimal (Microvesicular)
Kidney	Glomeruli changes	None	None	None
	Proximal tubule changes	None	Mild secretion	Minimal secretion
	Congestion	None	Mild	Minimal
	Inflammatory cell infiltration	None	None	None
	Interstitial fibrosis	None	None	None

## Data Availability

The original contributions presented in this study are included in the article. Further inquiries can be directed to the corresponding author.

## Abbreviations

ADH	alcohol dehydrogenase
ALDH	aldehyde dehydrogenase
ALT	alanine transaminase
AST	aspartate transaminase
BUN	blood urea nitrogen
CHOL	cholesterol
CREA	creatinine
GSH	glutathione
H&E	hematoxylin and eosin
IACUC	Institutional Animal Care and Use Committee
NAFLD	non-alcoholic fatty liver disease
RA	rubeanic acid
SOD	superoxide dismutase
TRIG	triglycerides
